# Association between Maternal Anaemia and Postpartum Depression in an Urban Sample of Pregnant Women in Iran

**DOI:** 10.3329/jhpn.v31i3.16832

**Published:** 2013-09

**Authors:** Azita Goshtasebi, Mozhgan Alizadeh, Samira Behboudi Gandevani

**Affiliations:** ^1^Family Health Research Group, Health Metrics Research Center, Iranian Institute for Health Sciences Research, ACECR, Tehran, Iran; ^2^Imam Hospital, Sari, Iran, Research Institute for Endocrine Sciences, Shahid Beheshti University of Medical Sciences, Tehran, Iran; ^3^Reproductive Endocrinology Research Center, Research Institute for Endocrine Sciences, Shahid Beheshti University of Medical Sciences, Tehran, Iran

**Keywords:** Anaemia, Edinburgh Postpartum Depression Scale, Haemoglobin, Postpartum depression, Iran

## Abstract

The aim of this prospective study was to determine the relationship between anaemia during pregnancy and postpartum depression. Two hundred eighty-one non-anaemic mothers with singleton and low-risk pregnancy and no history of antidepressant-use were studied. Demographic and reproductive data at week 20 were obtained. Mothers were followed up and haemoglobin (Hb) was checked at delivery. Iranian version of Edinburgh Postpartum Depression Scale (EPDS) was completed 4-6 weeks after delivery. Mean age of the mothers was 26.6±4 years. The prevalence of postpartum depression according to EPDS was 5.5%. Binary logistic regression analysis showed that Hb <11 g/dL at delivery would increase the chance of postpartum depression (OR 4.64; 95% CI 1.33-16.08). The results show that diagnosis and treatment of physiologic factors, especially anaemia, would reduce the risk of postpartum depression.

## INTRODUCTION

Postpartum depression (PPD) is a major episode that most often emerges within 6 to 12 weeks after delivery (1,2). The prevalence of postpartum depression is about 10-15%, depending on the diagnostic criteria, timing of screening, and screening instruments used (3). Apart from affecting mental health of a mother, it also disturbs family relationships and emotional and cognitive development of a child (4,5).

A number of psychosocial risk factors have been identified that entail the development of PPD but few studies have recognized physiologic variables contributing to PPD (6). Recently, the role of iron-deficiency anaemia (IDA) has been considered. Prevalence of IDA during pregnancy is around 7.5%, and recovery of iron stores in the postpartum period is often delayed in women who experience IDA during pregnancy (6,7). Behavioural symptoms associated with anaemia in adults include changes in cognition, emotions, irritability, apathy, fatigue, depressive symptoms, and hypoactivity (7,8).

Alterations in thyroid hormone metabolism (6), changes in the neurotransmitters function (9), and reduction in some inflammatory cytokines, such as interleukin 2 in iron deficiency, might be underlying causes for the development of PPD (10-12).

There is a relative lack of information regarding the influence of maternal anaemia on postpartum depression. The aim of our study was to determine the relationship between anaemia during pregnancy and postpartum depression.

## MATERIALS AND METHODS

### Participants

For a significance level of 0.05 and to find a 10% prevalence of postpartum depression, the number of subjects required was 216. Two hundred eighty-one healthy pregnant women were included in the study from February to December 2009. The study population consisted of primipara women aged 18-35 years with normal BMI (19.8-26) with singleton pregnancy. Women with iron-deficiency anaemia (defined as Hb <10.5 g/dL), history of chronic disease or other diseases known to interfere with iron metabolism, history of antidepressant-use, stressful life-events, consumption of alcohol, cigarette smoking, or drug-abuse, were not recruited.

### Setting

The participants were registered at prenatal clinic of Imam Hospital at Sari, a coastal city located in north of Iran.

### Design

During a longitudinal study, low-risk pregnant mothers with parity ≤2 and singleton pregnancy were enrolled in the first prenatal visit at 14-20 weeks of gestation. Demographic and obstetric data and laboratory test results were collected. The eligible mothers in their first prenatal visit were examined for iron-deficiency anaemia, and women with Hb <11 g/dL and ferritin <15 mcg/dL were not recruited. All mothers received routine prenatal care (including standard vitamin and mineral supplementation) and followed up during the prenatal period till delivery. Maternal blood haemoglobin and ferritin concentrations were determined at birth. According to the CDC reference values for haemoglobin (Hb <11 g/dL in the third trimester for anaemia), the women were divided into anaemic and non-anaemic groups. They were followed up till 4-6 weeks after delivery. Iranian version of the Edinburgh Postpartum Depression Scale (EPDS) was employed 4-6 weeks after delivery. The EPDS score 13 was used as cutoff point for screening depression in the mothers under study.

### Drop-outs

Two mothers were excluded (one with severe pre-eclampsia and pregnancy termination before week 34, one having idiopathic thrombocytopenic purpura), and 25 (10%) mothers were not accessible and did not complete the EPDS. Demographic and obstetric characteristics of these mothers did not differ from the others.

### Measures

Iranian version of the Edinburgh Postnatal Depression Scale (EPDS) was used as the screening tool for postpartum depression. This scale was used successfully in previous studies on postnatal depression in Iran (13,14). In general, the Iranian version of the EPDS was found to be acceptable to almost all women. Cronbach's alpha coefficient was found to be 0.77. Validity as performed using comparison among known groups showed satisfactory results. The questionnaire discriminated well between subgroups of women differing in mode of delivery in the expected direction. The factor analysis indicated a three-factor structure that jointly accounted for 58% of the variance. A cutoff score of 13 provided 95.3% sensitivity and 87.9% specificity for clinical depression (13). Maternal Hb values were determined by the cyanmethohaemoglobin method. A complete blood count was done using an automatic cell counter (T890, Coulter), and serum ferritin was assessed by radioimmunoassay (Gamma Counter System, Kontron).

### Data collection

Data were collected in the prenatal clinic and labour ward of Imam Hospital in Sari, Iran. A venous blood sample at the time of delivery was obtained and was immediately assessed for anaemia. Then, mothers were followed for 4-6 weeks after delivery.

A specific room in prenatal clinic (which is also the postnatal care clinic) was allocated for this job, and every mother was invited to the room and asked to fill up the questionnaire. A trained midwife was in charge of all data-collection process. She was present in the room to help mothers who had difficulty in reading or understanding the questionnaire.

### Ethics

The study was approved by review board of the Iranian Institute for Health Sciences Research (ACECR), Tehran, Iran. An informed consent for scientific use of the data was obtained from each subject.

### Statistical analysis

All statistical analyses were performed using SPSS (version 11.5) for Windows (SPSS Inc., Chicago, IL). Based on Hb concentration at delivery, participants were divided into anaemic and non-anaemic groups, and all comparisons were performed accordingly. Mothers were grouped into three educational levels based on the completed years of academic education. The results were presented as mean±SD. Comparisons between groups were performed using unpaired *t*-test, χ^2^-test, and binary regression. Statistical significance was set at the 95% level (p<0.05).

## RESULTS

Overall, 254 women were included in the final analysis. There were no obstetric complications in the final sample studied. The sociodemographic and obstetric characteristics of the study women are shown in Table 1.

The women in two groups were not significantly different in terms of age, job, education, BMI, and gestational age at delivery. The mean age of mothers was 26.6±4.0 years, and the mean schooling of formal education was 9.24±3.5 years. Most of the study women were housewives (97.6%); 14 of 254 women (5.5%) had postpartum depression according to EPDS. The incidence of anaemia (Hb <11 g/dL) at delivery was 14% without hypoferritinaemia. Table 1 shows the probability of PPD at 4-6 weeks postpartum in anaemic and non-anaemic mothers at delivery. The mean values of ferritin were not different in anaemic and non-anaemic mothers. Binary logistic regression analysis showed that Hb <11 (OR 4.64; 95% CI 1.33-16.08) at delivery and secondary level of education (OR 6.76; 95% CI 1.10-41.16) would increase the chance of postpartum depression (Table 2).

## DISCUSSION

Anaemia, as a physiological cause of PPD, has recently received attention from scholars in both the fields. Findings of this study and other similar works could provide evidence for this.

The aim of the study was to evaluate the effect of iron-deficiency anaemia on postpartum depression. However, since the study sample was derived from a low-risk population and according to national iron supplementation programme, all pregnant mothers should receive iron supplements during pregnancy, regardless of ferritin or Hb levels; no mothers at the time of delivery had iron-deficiency anaemia (low haemoglobin and ferritin values). Our study demonstrated 5.5% prevalence of postpartum depression, which is much lower than the figures described previously (2,3,14,15). This may be due to the different tools and the cutoff points used by others. In the present study, we used Iranian version of the EPDS because it has been proven as an acceptable, reliable, and valid measure of postnatal depression among women in Iran. Also, we used a cutoff value of 13 to signify depression. This cutoff has been shown to have high sensitivity and specificity in Iran (14). Moreover, the selected sample was very healthy and of low risk for any complication, including postpartum depression.

**Table 1.** Sociodemographic and obstetric characteristics of the study women

Characteristics	Anaemic	Non-anaemic	p value
Age (years)			0.79
<21	2	16	
21-30	30	171	
>30	4	31	
Age^1^: mean (SD)	25.50 (4.37)	26.11 (4.03)	0.40*
Education			0.88
Primary	8	46	
Secondary	27	162	
University	1	10	
Education^2^: mean (SD)	9.44 (3.59)	9.21 (3.49)	0.57*
Housewives: (%)	97.2	97.7	0.87**
BMI^3^: mean (SD)	23.19 (1.78)	23.54 (1.72)	0.27*
Gestational age at birth^4^: mean (SD)	273.58 (1.313)	274.26 (1.148)	0.64*
Caesarean delivery: (%)	50	59.6	0.27**
Pregnancy weight gain^5^: mean (SD)	12.87 (2.10)	12.80 (1.97)	0.80*
Newborn sex (male): (%)	55.6	49.1	0.47**
Hb^6^ at delivery: mean (SD)	11.57 (0.67)	11.93 (0.71)	0.004*
Ferritin^7^ at delivery: mean (SD)	58.6 (43.8)	55.6 (35.5)	0.74***
EPDS score <13: (%)	13.9	4.1	0.018*

^1^Years

^2^Years of completed academic education

^3^kg/m^2^

^4^Days

^5^kg

^6^g/dL

^7^mcg/dL,

*t-test

**χ^2^-test

***Mann-Whitney test

**Table 2.** Results of logistic regression analysis with the odds ratios of postpartum depression for each risk factor adjusted for other variables in the model

	Adjusted OR	95% CI
Age^1^		
<21: 18**	1	
21-30: 201**	0.40	0.02−7.29
>30: 35**	0.45	0.05-3.78
Educational level		
University: 54**	1	
Secondary: 189**	6.76	1.10-41.16*
Primary: 11**	3.70	0.51-26.37
Type of delivery		
NVD^2^: 106**	1	
Caesarean^3^: 148**	1.81	0.55-6.01
Male: 127**	1	
Female: 127**	0.61	0.19-1.94
Gestational age at delivery^4^		
=37: 38**	1	
<37: 216**	2.93	0.33-25.43
Hb at delivery^5^		
=11: 218**	1	
<11: 36**	4.64	1.33-16.08*

^1^Years;

^2^Normal vaginal delivery;

^3^Caesarean section;

^4^Weeks,

^5^g/dL;

*p<0.05;

**Distribution of sample in each category

Research suggests several psychosocial risk factors for postpartum depression but few from cited studies examined the relationship between physiological factors, specifically anaemia and postpartum depression. This topic is a relatively new one in the field. There is a large body of literature on postpartum depression and its risk factors but these did not focus on physiological factors, such as anaemia to the desired extent.

There are some controversial studies in this field. Wolf and colleagues (15) did not find an association between maternal iron status and depression symptoms at 1 year postpartum in a large sample of mothers from Chile and Costa Rica. Corwin and co-workers (6) demonstrated a significant relationship between anaemia and depressive symptoms in the postpartum period.

The results of our study suggest that Hb <11 g/dL at delivery, but not iron deficiency, would increase the chance of postpartum depression. The wide confidence interval might be explained by small sample-size or homogeneous low-risk mothers we studied. Based on routine prenatal care at the national level, all mothers received iron supplement during pregnancy, and we did not detect any hypoferritinaemia in our samples. This suggests that anaemia regardless of iron status might affect emotions in postpartum period. Considering the negative consequences of depression on the mother, her offspring and whole family, diagnosis and treatment of anaemia should be an essential part of maternity care during pregnancy and after delivery.

This study is original in that it is one of the first studies in Iran to report on the association between anaemia and postpartum depression.

### Limitations

Our study had a few limitations that should be considered. As it can be seen from Table 1, the study sample came from an urban population of low socioeconomic status. We excluded all the mothers who had stressful life-experiences, history of using antidepressant drugs and mental or physical disorders. There were no maternal or neonatal complications or birth of even LBW neonates. Most mothers were young and had their first or second pregnancy. However, we did not assess the social support to the study mothers. Several studies showed that the lack of social support can increase the incidence of PPD. Therefore, we tried to remove as many as possible factors influencing the level of stress and receiving support from the family and society.

### Conclusions

Hb concentrations at delivery were used as an indicator of maternal anaemia during early postpartum period. While some studies showed that the concurrent Hb values might be more practical, others showed that anaemia at delivery usually continues during postpartum period. Although the sample-size was relatively small, the relationship between anaemia and PPD was evident in the present study.

## ACKNOWLEDGEMENTS

This study was funded by Iranian Institute for Health Sciences Research (ACECR). We would like to thank all the mothers who participated in this study. We also wish to express our gratitude to staff of maternity care clinic in Imam Hospital.
